# Indigenous trans-systemics: changing the volume on systems

**DOI:** 10.1007/s11625-023-01330-3

**Published:** 2023-05-25

**Authors:** Don G. McIntyre, Geneva A. Cloutis, Dan McCarthy

**Affiliations:** 1 Dhillon School of Business at the University of Lethbridge, Lethbridge, Canada; 2 School of Environment, Resources and Sustainability at the University of Waterloo, Waterloo, Canada

**Keywords:** Critical systems thinking, Humility, Indigenous trans-systemics, Systems thinking, Trans-systemics

## Abstract

This paper emerged as a result of Anishinabe and non-Indigenous scholars discussing the basic principles behind systems thinking. By asking the question “what is a system?”, we uncovered that our very understanding of what makes a system was vastly different. As scholars working in cross-cultural and inter-cultural environments, these differing worldviews can create systemic challenges in unpacking complex problems. Trans-systemics offers language to unearth these assumptions by the recognition that the dominant, or “loudest”, systems are not always the most appropriate or equitable. It goes beyond critical systems thinking to identify that tackling complex problems requires the recognition that there are multiple, overlapping systems and worldviews at play. We identified three key takeaways from Indigenous trans-systemics for socio-ecological systems thinkers: (1) trans-systemics is a call to humility, asking us to critically examine our patterns of thought and behavior; (2) by exploring humility, trans-systemics allows us to move past the autopoiesis of Eurocentric systems thinking to consider interdependence; and (3) to utilize Indigenous trans-systemics, we need to fundamentally reconsider how we understand the systems around us and bring in outside tools and concepts to enact meaningful systems change.

## Context/positionality

This paper began with a conversation between two colleagues, one is an Anishinabe legal scholar and the other is a non-Indigenous, systems scholar mostly of Irish descent, about their respective research programs. The conversation eventually centered on a couple of seemingly simple questions, “what is a system?” and “if an observer is not there to observe a system, does the system still exist?”. Of course, these are not simple questions, and we did not necessarily answer them, but the discussion that flowed around these questions began to reveal more about the assumptions and worldviews of the two colleagues than it did about systems. It surfaced an array of disciplinary- and culture-based biases, blind spots or unconscious material that had to be made conscious to have a more fruitful discussion about the complex epistemological and ontological issues that were really at the heart of this discussion. And it was the concept of trans-systemics that offered the two colleagues a framework and a language to bring these unconscious biases into consciousness and enrich the discussion about systems. Trans-systemics is a legal term and, generally, refers to a demand for greater inclusion of the French language and civil code within the Canadian legal landscape. The expansion of this language and jurisprudential discourse into other areas ties into the authors’ teachings regarding how to view and articulate Indigenous systems. It is our contention that Indigenous systems should be viewed without the ‘silo-ing’ that plagues much of conventional thinking within the academy. Indigenous ways of knowing provide a much more permeable perspective when it comes to how to view ones’ area of expertise. For these reasons, this paper is an invitation to use Indigenous trans-systemics (Battiste and Henderson 2021) to expand the voices that must be heard in studying, engaging, and transforming the systems we inhabit.

The prefix ‘trans’ transforms the root word to which it is attached. Originating from the Latin trans or meaning ‘across’, the Oxford dictionary defines it this way: across, beyond, through, and surpassing. With this in mind, trans-systemics is an attempt to explore systems beyond the dominant, monocular understanding of systems, toward a poly-systemic perspective. This paper serves to describe key concepts related to systems thinking and complexity, to offer a critique of its use in contexts with multiple worldviews, and to introduce the idea of trans-systemics in both Eurocentric and Indigenous contexts.

This paper looks at the idea of Indigenous trans-systemics and how it can benefit all work in the field of social–ecological systems. Indigenous trans-systemics is a bricolage of trans-systemics as articulated through the work at the McGill University Law School since 1999 and the struggle of Indigenous people to have their systems recognized within Canadian systems since first contact. The trans-systemic approach to legal education facilitates the integration of both common law and civil approaches in a bilingual, bijural, and dialogic manner. The McGill program aspires to legal pluralism. However, it does so with a decidedly Eurocentric approach to what is recognized as law

Canada provides a favourable context for the organic development of a trans-systemic approach to law. Transsystemia is a way to study and understand law that goes beyond legal traditions…. a legal approach centered on a dialog between legal traditions, anchored in a pluralist and non-hierarchical method that celebrates the irreducible differences and similarities between various legal traditions….transcend[ing] the traditional dichotomies between civil law and common law. It also challenges the idea that civil law is better expressed in French in Canada, while the common law’s natural expression is in English. Finally, it relies on interdisciplinary approaches, thus drawing from other disciplines than law. (Emerich 2017)

Indigenous trans-systemics accepts this idea of how the West deems its laws should be understood but additionally demands the recognition and incorporation of Indigenous ways of knowing within Canadian systems. Recognizing that Indigenous legal systems look beyond themselves to define and understand the rules of law, trans-systemics from an Indigenous perspective must look to other systems and knowledges to create a new orientation that is both Eurocentric and Indigenous simultaneously. Having its genesis in these two intersecting areas, which will be explored later in the paper, allows Indigenous trans-systemics to serve two functions. First, it continues with the struggle to provide voice for Indigenous Peoples in Canada. This voice continues to be denied or discounted in the sectors of business, government, social, economic, science, and health; to name a few. Its second role is to engage mainstream thinking by introducing new-to-the-status-quo ideas, concepts, approaches, and ways of being that are tried and true within Indigenous systems. This will expand the grasp, reach, and effectiveness of all sectors and systems that continue with a Eurocentric knowledge base.

While we are not the first people to discuss the concept of trans-systemics, Legal trans-systemics or even Indigenous trans-systemics, we believe what we offer in this paper is a framework that social–ecological systems scholars and practitioners can use to elevate their discussions, learning, research, and application of systems thinking in contexts that involve very different worldviews coming together and approaches to ensure that this is done in a good way. In many ways, this paper tracks the learning journey or chain of logic that enabled these two colleagues to come to a place of understanding around their differing views of systems. We offer this to colleagues as a way to begin or continue the work of thinking in systems, thinking about systems, and thinking about thinking within systems that will enable a more humble, honest, and hyper-reflexive approach to exploring and applying social–ecological systems thinking in difficult or contentious contexts (Machado de Oliveira 2021). We begin our discussion of trans-systemics with a story offered by our storyteller and lead author Don McIntyre.

### Trans-systemics and Nahnabush as transformer

For the Anishinabe, all things are connected through story. Story to explain our legal system, our education system, our environmental system, our cultural system, and how they are all connected through a system of narration. There is a story of the Anishinabe Trickster, Nahnabush, and an encounter that started with the Winged Nation on the shores of Lake Timiskaming.

Two things to know about Trickster, the first is that their stories teach lessons; sometimes by doing the right thing and sometimes by doing the wrong thing. The other thing to know is that Nahnabush, like all Tricksters, held many gifts and powers. One of these gifts was the ability to transform and change his shape. In this story, Nahnabush and his Grandmother were sitting on the shore of Lake Timiskaming. It was the time of the Winged Nations’ migration, and so, the lake was covered with the Winged travelers. The Winged Ones were spending days on the lake resting, gathering, and preparing for the long journey through the Southern Threshold.

As he sat with his Grandmother, looking out at the Winged Ones covering the Lake, Nahnabush asked, “are you hungry?” Grandmother suggested that she could eat. And they agreed that she would start a fire and he would get them some dinner. Nahnabush walked to the edge of the water, and as he stepped in, he transformed his shape just enough to allow him to breathe underwater.

Looking up from the bottom of the lake with his feet firmly planted on the ground, he saw how the fish swam over there, and how the plants grew over there. He became aware that the Lake was colder down here. Looking up to the surface of the lake, Nahnabush noticed that rather than calmness among the Winged Ones that he perceived from the Shore, here, everything was madness. The ducks and geese paddled their feet wildly attempting to stay afloat. Tricking them with an Inter-tribal song, Nahnabush got the Winged Ones to move in sync. This allowed him to wrap a magical rope around all their feet. Once he had all their feet bound up in the rope, he walked out of the water, transforming so he could breathe on land, Nahnabush pulled up the rope that was tied to all the feet. When he did this, the Winged Ones flew up taking Nahnabush with them.

They traveled up 10, 100, 1000, and 10,000 feet into the sky. And there, Nahnabush could see the Four Directions that Humans had traveled. The Eastern, Southern, Western, and Northern Thresholds. He got dizzy spinning from the rope. He realized, he could fly, just like Goose; so he cut himself free of the rope and the Winged Ones. Soaring through the air, Nahnabush became aware that he was about to hit the lake…HARD. But just before he crashed into the water, he transformed into a Pike and entered the water easily.

Under the water, as Pike, Nahnabush was aware of a very different environment in Lake Timiskaming. He did not look for the ground. Grounding was unnecessary as his perspective was transformed as he became part of the Lake. He forgot about his home, his people, his Grandmother, and his time. He knew only the Lake and his obligations as Pike. He took on this role; without knowing.

This Anishinabe understanding of systems thinking addresses a primary disjoint in our conversations regarding systems theory. What is the role and responsibilities of the observer? The outsider? Can the outsider truly observe or articulate another’s system?

## Introduction

We can imagine our world as a system, a set of components bound together by relationships and interactions that, in the relationship, result in their own, emergent behavior. Within that system, there are an almost infinite number of smaller systems, which interact and co-exist with one another. Most of us are taught and trained to understand a single “set” of systems which we use to make sense of the world around us. These include, for example, legal, economic, energetic, social, and environmental systems, etc., which frequently overlap with one another but offer tools and lenses with which to understand the world around us. Researchers and scholars have come up with terms to define and describe systems, which offer powerful tools to explicitly detail the systems around us. However, these terms were conceptualized based on certain perspectives, which in turn were based on certain systems. Being deeply engrained in particular systems means that it is often difficult or impossible to imagine that other systems are also at play simultaneously that are beyond our comprehension.

**Fig. 1:**
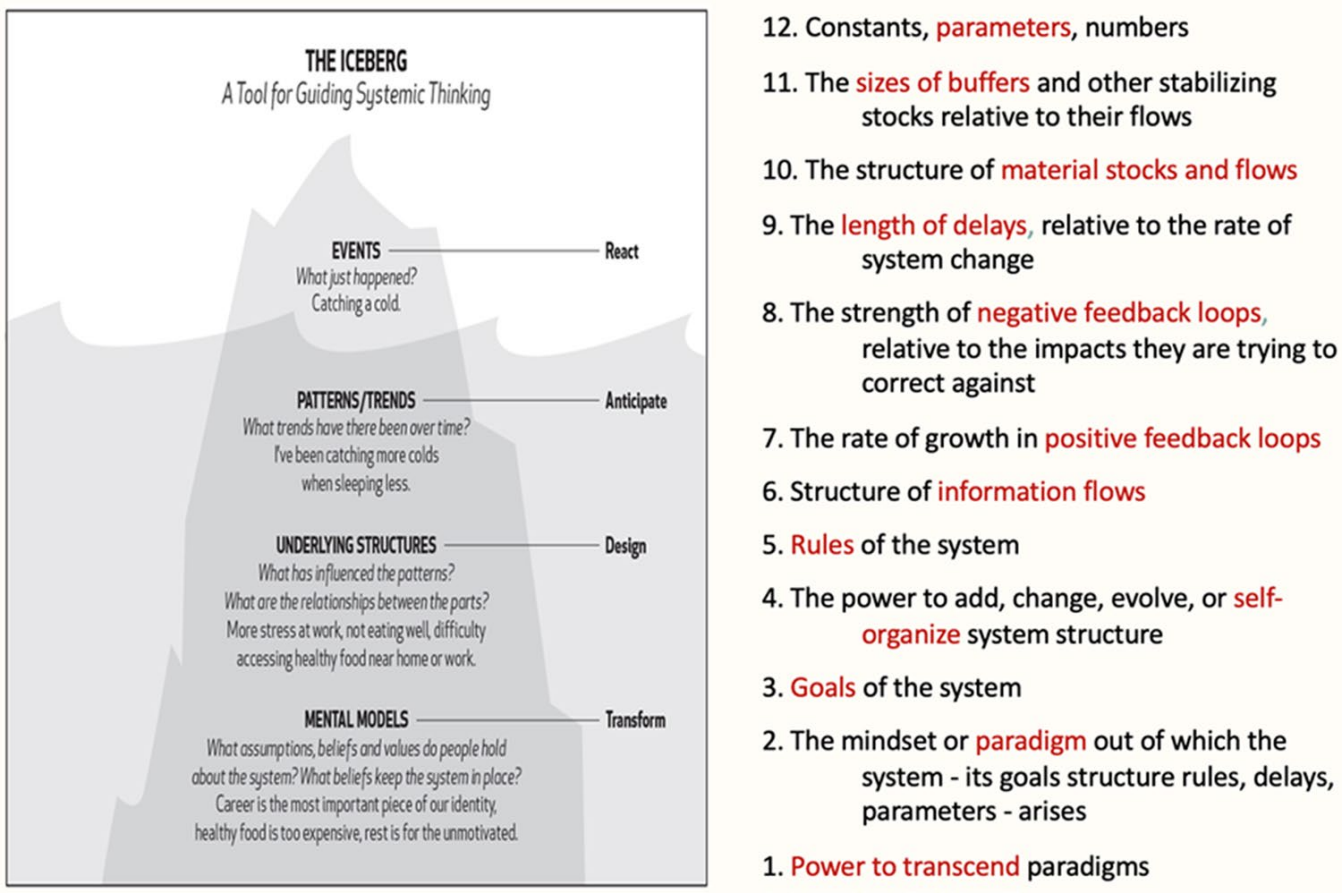
Iceberg model as a tool for systems thinking, adapted from Senge (1997), and the Leverage Points, adapted from Meadows (1999)

Two existing, systems-based frameworks for thinking that elucidate the premise behind Indigenous trans-systemics are the related ideas of the “Iceberg model” often used by systems thinkers such as Senge (1997), as well as Donella Meadows’ leverage points (see Fig. 1). These related frameworks demonstrate the importance of systems thinking as well as the need to move down the iceberg to the underlying mental models, as well as Meadows’ notion of trans-paradigmatic thinking, or the power to transcend paradigms, as one of the most potent leverage points we have. This for us is the rationale for describing Indigenous trans-systemics, but this connection we recommend be explored further in subsequent research.

If we imagine all systems as exhibiting a particular sound or noise, we often have the volume turned up so loudly on one system that we cannot hear the other systems.

**Fig. 2:**
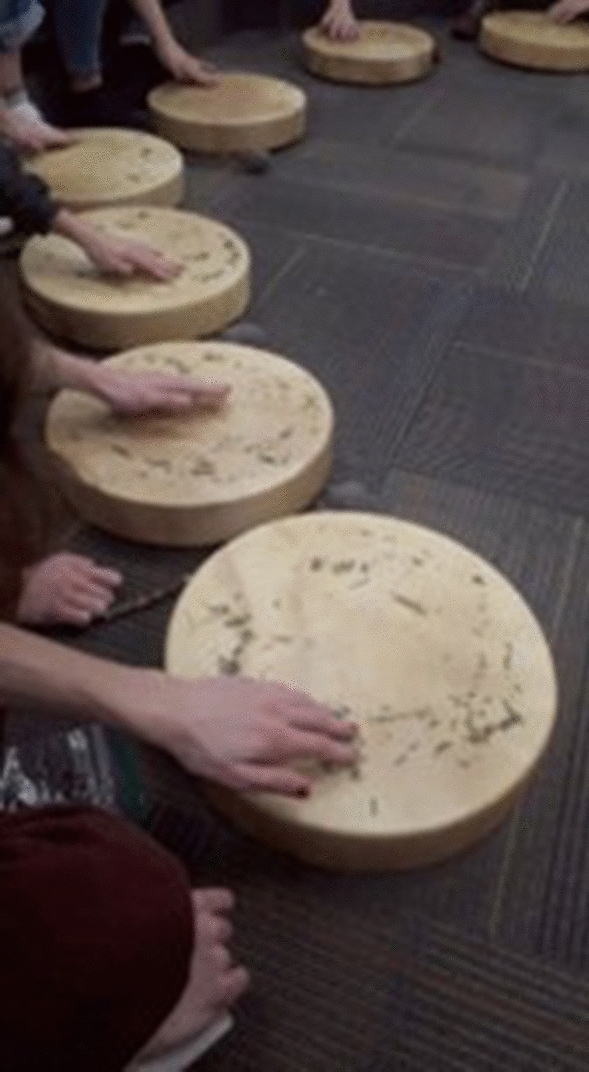
Hand drums placed on the floor create a muffled sound

**Fig. 3:**
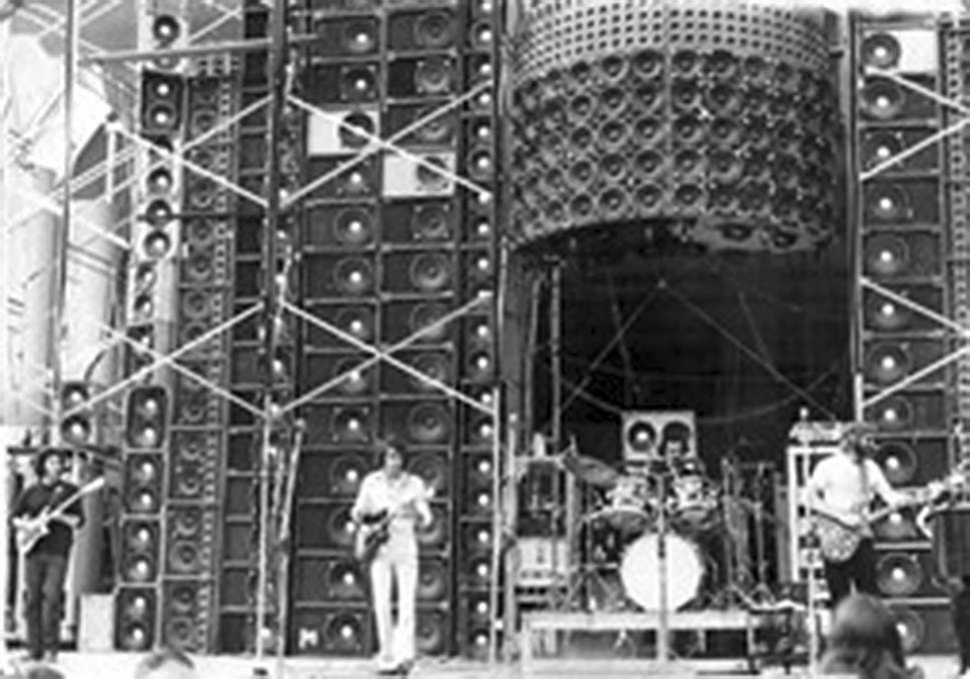
Amplifiers at a concert are purposefully turned up so loud that no other sounds can be heard. The Base drum can drown out all unwanted noise

**Fig. 4:**
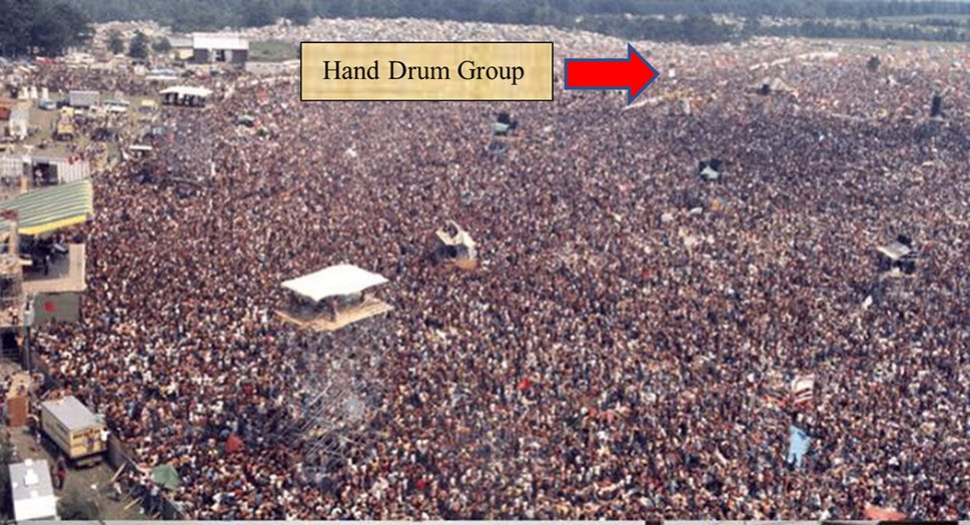
This dominant system will drown out any other sound within miles, no matter how important the message

An Indigenous hand drum can reach say 50 people (Fig. 2). Drumming with others and you could reach 5 times that number. This has been proven at gatherings for millennia. But put that drumming group into the center of the audience at a heavy metal rock concert and they would go unheard (Figs. 3 and 4). Their voices swallowed up by the heavily amplified message of the band on stage. Simply because we are able to amplify the band on stage, does not necessarily mean that the music or the message is more important for society. Trans-systemics, in essence, is the ability to turn the noise down on the dominant system to hear what else is going on. That is, to enable a level of discernment in the use of different ontological and epistemological perspectives, as opposed to defaulting to the loudest perspective. The authors recognize an artificial binary of Indigenous/non-Indigenous, Western/Indigenous, Settler/First Peoples, and the like. This is a shorthand representing hundreds of nations (both Western and Indigenous) that has been created and imposed on the discourse. The aim of Indigenous trans-systemics is to move through these binaries and false dichotomies to more inclusive conversations.

It seems that it should go without saying, but we will say it. Using the analogy of the drum group and the concert band, the concert-goers paid to hear the Headliners. This position comes with a level of prestige. The stage is set up to their specifications. The opening acts work around their equipment. Backstage is arranged for their wants and desires. The dates of the concert which brought all these people together are set by the calendar of the Headliners. They control placement and positioning of the speakers—and the volume. This is power that comes from being in the privileged position of being the Headliner. Even if the Drum Group had been coming to this field to sing for thousands of years, the Headliners make the decisions about this territory for a few days, and then, they leave. Without regard for the garbage left, the plants destroyed, or how the animals and other organisms that come under threat to their habitat struggle to return to pre-concert numbers. If the field cannot be returned to its pre-concert state fast enough, biodiversity of the habitat may be lost. However, the Headliners are unaware of any of this. From their perspective, the sound check went well, the concert was ‘a blast’, they move to the next venue, and the band plays on. Indigenous trans-systemics asks the Headliner to consider all the systems that are attempting to run parallel to their ‘gig’. Not just the Drum Group but every other organism within many concurrent systems at play using their power and privilege to turn down the volume of the amplifiers to give the organisms within those other systems a chance to survive.

In more technical terms, scholars from a wide variety of disciplines, including physics, mathematics, chemistry, biology, ecology, and operations research, have developed a growing array of formal theories that have helped us to better understand the structure and dynamics of complex adaptive systems (Thom 1975; Prigogine and Stengers 1984; Maturana and Varela 1987; Lorenz 1993; Kauffman 1995). These formal theories have formed the foundations for more robust approaches to a wide variety of fields including, but not limited to, ecosystem dynamics and resilience, environmental management, governance (Gunderson et al. 1995; Gunderson and Holling 2002; Folke et al. 2005), organizational behavior (see, for example, the work of Peter Senge), as well as emerging approaches to innovation and systems transformation (Olsson et al. 2004; Westley et al. 2011), and offer opportunities to tackle some of the world’s more complex problems (Olsson et al. 2014; Sharpe et al. 2016). However, like any approach, there are limitations to their applicability, particularly in contexts where there are multiple, inter-related systems at play. One of the largest self-referential challenges within systems thinking is the requirement to identify, describe, and resolve all entities of the “system” through the singular lens of the system. These self-imposed standards require everything to fit within the definitions created by the discourse, and we are forced to dismiss anything that cannot fit or force it into a definition accepted by systems thinking. Despite acknowledging that conventional systems thinking requires us to shortchange countless systems, we accept it as the cost of allowing the discourse to continue. Moreover, the definition of a system depends largely on whoever is describing “the system”, based on their worldviews, lenses, and experiences interacting with the world. Different worldviews and cultures may understand “systems” in entirely different ways than the perspectives under which systems thinking and complexity were postulated. This paper calls into question the standard understanding of the notion of a “system” and borrows from the Eurocentric legal landscape to introduce the notion of trans-systemics as a solution to working around this challenge of systems and as a framework for fostering more productive dialogue about complex social–ecological systems from very different understandings of what the system is, could be or should be.

We ask indulgence as we present to you a new way for social–ecological systems thinkers to think. We will share some of the beginning insights between the authors by addressing the ideas surrounding first principles and how they differed from our divergent perspectives. It was a realization when the three authors began talking about first principles that we were talking about three very different narratives. We will first raise some high-level philosophical and epistemological ideas around systems, followed by a description of some basic systems concepts that require critical examination to unpack trans-systemics. We will then discuss these systems-based concepts with the intent of identifying the need for a Trans-systemic perspective for addressing the limitations associated with defining systems from a single, hegemonic perspective. We will then introduce Critical Systems Thinking as a logical extension of systems thinking, which tackles some of the “simpler” challenges that arise. We will then critique this perspective to further justify the need for the inclusion of trans-systemics in systems thinking, especially in the context of complex, cross-cultural problem domains such as reconciliation.

## Psychological roots of systems

When we enter into a discussion of the nexus of Indigenous knowledge and complex systems thinking, we inevitably end up on the landscape of philosophy, particularly ontology and epistemology. On this landscape, we most often point to distant points on the landscape, divided by vast, almost endless, tracts of wilderness between the very different (and often quite arbitrary) landmarks of “Traditional Knowledge” and “Eurocentric Science”. Alternatively, some authors try to build bridges and roads between these landmarks through simple comparisons, noting their similarities. This paper offers a framework to help remind, particularly social–ecological systems thinkers, that the philosophical landscape between these landmarks is a very complex one that is deeply influenced by many factors, including the view of the position on the landscape we currently hold, our individual ability to see and hear the landscape, and even the way our psyches and brains process that information. Other scholars have provided important and precedent-setting approaches for approaching research in these difficult and complex inter-cultural spaces, including but not limited to, decolonizing methodologies (Smith 1999), relational accountability (Wilson 2008), Indigenous Methodologies (Kovach 2009), Ethical Space (Ermine 2007), Two-eyed seeing (Bartlett et al. 2012), Hospicing Modernity, (Machado de Oliveira 2021), and Relational Systems Thinking (Goodchild 2021). Many social–ecological systems thinkers and resilience scholars have worked in these difficult, interstitial spaces, and have discussed the importance of, for instance, “Multiple Evidence Based” approaches (Tengö et al. 2017; Lam et al. 2020). Trans-systemics represents a reflexive complement to these approaches, inviting participants to reflect on their own forms of bounded rationality and which systems’ volumes may be too loud, and what other systems may exist that need to be turned up.

Attempting to work on this landscape not only requires one to try to map it, but also to note your perspective, projection, psychological biases, and finally, to reflect on how the landscape, your projection of it, and your psychological perspective all influence each other. Trans-systemics, as a framework for working in difficult, interstitial spaces, helps reinforce Machado de Oliveira’s (2021, quoting Stein) 4 H’s—humility, honesty, humor, and hyper-reflexivity. This kind of inner work, or inner development, is critical for a Trans-systemic approach. This work is characterized by an invitation for individuals working in complex social–ecological systems to reflect on their individual histories, their identities, and their privileges and identify entrained, unconscious patterns of thought and behavior that often, inadvertently reinforce existing structures and power dynamics. The challenge here, as Machado de Oliveira (2021) points to, is to uncover these unconscious patterns of thought and behavior, interrupt them, and then practice discernment with reference to alternative patterns. This kind of psychoanalytic approach can help to release individuals from many forms of, what systems thinkers refer to as bounded rationality (see page 19), and allow them to uncover systemic power dynamics and see new opportunities for systems change.

Systems thinkers, such as David Snowden, have noted that neuroscience highlights the fact that our brains are only able to pick up about 5% of the phenomena we might perceive and that we rely on entrained patterns of “first-fit” vs. “best-fit” pattern matches to solve problems. Neuroscientist McGilchrist noted that, even on a neurological level, we have “entrained patterns” which are reinforced by the resultant institutions and inhibit our ability to “see” or “hear” messages from the landscape in particular ways. On the psychological level, depth psychologists have long noted that the ego, the part of the psyche that has self-awareness and that we use to process most of the stimuli around us, is painfully limited in its ability to process the cacophony and complexity of stimuli we face. As a result of our child experiences, family context, socio-economic status, culture, language, and other factors, we develop complexes, or patterns of thought and behavior, that take over when we are faced with complex or unfamiliar stimuli.^1^

We “see”, “hear”, and truly experience only a small subset of structures and dynamics of the systems around us. While our brains and our psyches, limited as they may be, have allowed for the increase in complexity that we see around us, as well as the institutions that enable us to maintain and innovate within the systems around us, they too have “complexes” and an “unconscious”, that not surprisingly are an emergent property of our repeated behaviors. Trans-systemics represents a therapeutic framework for systems thinkers to process the blind spots, or unconscious biases, present in the shadows of Eurocentric science and systems thinking.

Yunkaporta (2019), in his book Sand Talk, uses the Australian Aboriginal yarn about the Emu to describe one of the fundamental human problems that has led to everything from simple arrogance and narcissism to colonization, racial inequalities, wealth disparities, and the abuse/misuse of the earth:

Emu is a troublemaker who brings into being the most destructive idea in existence: I am greater than you; you are less than me. This is the source of all human misery. Aboriginal society was designed over thousands of years to deal with this problem. Some people are just idiots—and everybody has a bit of idiot in them from time to time, coming from some deep place inside that whispers, “You are special. You are greater than other people and things. You are more important than everything and everyone. All things and all people exist to serve you.” This behavior needs massive checks and balances to contain the damage it can do. (Yunkaporta 2019, pp. 26–27)

Trans-systemics is a reminder of the yarn about Emu as well as the Anishinabe story of Nahnabush. The systems we see and the approaches we have developed to understand them are amazing, have led to amazing new knowledge, and have allowed us to do amazing things, but they are not the only systems that can be seen just as they are not the only ways to see and experience them. Trans-systemics is also a reminder to not simply try to incorporate or integrate other ways of knowing or being into ours, it is an invitation to question, on the most foundational, philosophical level, to examine the Emu in all of us individually and in our institutions.

## Systems thinking

To properly dissect the Eurocentric understanding of systems, it is important to consider some of its foundational elements. At its core, a system is more than one component, that are brought together and transformed to function in an entirely new way that the component parts could not individually (adapted from Meadows 2008). Systems thinking emerged as a discipline in the 1950s as a way to understand these components and their interactions with one another and the environment (Checkland 2000). Systems thinking is interdisciplinary and combines many fields of knowledge and theory, including complexity, systems dynamics, and network theory (Hargreaves and Podems 2012). Systems thinking is a mode of reasoning and a set of analytical tools, with strong roots in ecology, which is now used to understand ecological, economic, and social systems (Holling 2001). Systems thinking is used largely to understand multiple steady states and to assist in describing system dynamics through non-linear processes and feedback loops (Duit and Galaz 2008). All systems are comprised of components and sub-systems, and each system in turn is nested within larger systems (Cabrera et al. 2008; Ramalingam et al. 2008). All system components interact with one another, forming relationships that will change and dictate overall system behavior (Cabrera et al. 2008). To explain trans-systemics, we must first examine the concepts of system perspective and boundary as two of the most foundational elements to conventional systems descriptions.

### System perspective

Essential to any systems description is the perspective from which, and the purpose for which, the system is being described. Waltner-Toews et al. (2008) emphasize that one cannot describe a system without identifying who is describing the system and why. The perspective and purpose from which a system of interest is described dictate the choice of spatial, temporal, and conceptual boundary, and, in turn, dictate the components and structure that are included within the boundary. Perspective will dictate what the observer will see in the systems description and the insights that will emerge from the systems analysis. Any system under study will look different depending on who is viewing it (Cabrera et al. 2008). Understanding one’s perspective, or frame of reference, is critical when exploring systems as an analytical tool (Cabrera et al. 2008). The ability to articulate and critique perspective is even more important when exploring Critical Systems Thinking, described later in this paper.

### System boundaries

The concept of a systems boundary is essential to defining a system of interest from a given perspective. Waltner-Toews et al. (2008) describe boundaries as an imaginary line which separates the components within the system from the external environment. It is an imaginary line, because it is a heuristic or conceptual tool, a creative construct, and highlights the fact that most (if not all) complex systems that we interact with are open systems, where energy, material, and information flow across the boundary. The purpose of the boundary, from a systems point of view, is to foreground the components/variables and their interconnections that are deemed to be of interest to the individual or group describing the system. In a social–ecological systems context, boundaries can be ecological (like a species range, or a watershed) or more social, such as political boundaries or an agency’s jurisdiction. Boundaries can often be difficult to define given their open and arbitrary nature, but they are important to define to explore and evaluate systems change. The concept of a system boundary is a key point of departure when trying to consider a Trans-systemic approach and also gestures toward the importance of a related concept for thinking about trans-systemics: bounded rationality.

### Bounded rationality

Bounded rationality has been described, in decision-making, as the fact that the “rationality of individuals is limited by the information they have, the cognitive limitations of their minds, and the finite amount of time they have to make a decision” (Simon 1955, p. 101). Or in systems thinking parlance, Meadows (2008, p. 108) described bounded rationality by stating that, “people make quite reasonable decisions based on the information they have. But they don’t have perfect information, especially about distant parts of the system”. Bounded rationality, in the context of trans-systemics, is a key point of departure for systems thinkers. Trans-systemics is ultimately a recognition of many layers of bounded rationality in our largely unconscious view of systems. When we draw a boundary around a system, we can forget that these boundaries are simply creative constructions, and so, they become unconscious and we assume a level of objectivity that may not exist. Trans-systemics is an invitation to systems thinkers to consistently and diligently ask questions about their boundary judgements and question what systems they may not be able to see or hear because of the unconscious blindness associated with the structure of our brains, our psyches, and the institutions and cultures that reinforce particular understandings, or “the volume” of a given system in relation to others.

## Thinking about systems: autopoiesis and critical systems thinking

In our discussions about systems and trans-systemics, it became clear that we needed to not only discuss systems thinking, that is, the nature and characteristics of systems but to think about systems and systems thinking itself. To foster this more ‘meta’ discussion, we used two theories/frameworks, Autopoiesis and Critical Systems Thinking, that point to meta-theoretical issues associated with systems thinking.

### Autopoiesis

Autopoiesis, meaning self-creation, was a theory developed by Maturana and Varela (1980) in the context of living systems to explore the qualifying factors of life itself. Maturana notes that the origins of the notion of autopoiesis can be traced back to 1960, when a first-year medical student asked the question, “what began three thousand eight hundred million years ago so that you can say now that living systems began then?”. Beyond this fundamental question, Maturana and Varela’s work goes on to address inter-related questions regarding the nature of reality (ontology) as we as humans experience it and the implications for knowledge and learning (epistemology) given that relationship. Autopoietic systems have networks which reinforce and regenerate the relationships required for their existence and define and are defined by those relationships and overall organization. Autopoietic systems will maintain themselves as distinct unities as long as they do not experience disturbances which affect their organization—a concept known as resilience (Westley and Laban 2012).

While autopoietic systems must maintain their organizational closure to maintain their identity, they must also maintain a relationship with their environment. Maturana and Varela (1980, 1987) describe this necessary interaction as “structural coupling”. Structural coupling is a process whereby the structure of the system and the environment both change or co-evolve as the result of iterative, mutual, non-destructive changes. Systems cannot be separated from their environments, even though systems thinking asks us to draw boundaries to define what is in the system and what falls outside. The above systems concepts have provided systems thinkers with the ability to understand the structure and dynamics of complex systems of various kinds, as well as the need to make explicit, and be critical of, assumptions associated with their choice of boundary (spatial, temporal, and conceptual) and their underlying perspective and purpose. However, there is still an analytical assumption that there is one system being described and this one system’s perspective is implicitly being reinforced. Critical Systems Thinking provides some resources to begin overcoming these assumptions, described in detail in the following section.

### Critical systems thinking

While the notions of perspective, purpose, and boundary do acknowledge that there are multiple ways to describe a system of interest, there are still several limitations in its use in contexts where multiple systems are overlapping with one another at the same time. Critical Systems Thinking concepts provide tools to explicitly uncover the limitations of our boundaries and perspectives and move systems thinking toward more emancipatory and inclusive ideas. The tenets of Critical Systems Thinking can be broken down into three broad ideas: critical reflection, pluralism, and emancipation (Midgley 2000).

Critical reflection posits that as we interact with systems, we must critique “the theoretical underpinnings, strengths and weaknesses of available systems models, tools and techniques” (Jackson 2000, p. 375). This must simultaneously take place with an element of social awareness, understanding how and when particular systems and system tools were developed. Canadian social innovation thought-leader, Tim Brodhead explains “Systems accomplish exactly what they were created to do.”^2^ The ‘Western System’ has created a series of interconnected systems that continually funnel the power and privilege into the Western system for which they were created. Consider how the ‘legal system’ addresses (either explicitly or implicitly) issues of education, governance, riparian rights, waterway, immigration borders, corporations, taxation, pollution, logistics, environmental protections, the list goes on and on leading to declarations of enforcement and punishment. This understanding of the power of law is taught in the education system which describes the powers of corporations and government and immigration policy, ownership over water, air, and land. The education system which describes rights to tax is paid for by the revenues generated through taxation. These sub-systems of the Western system make so much noise demanding that they have power and pointing to their privilege that no alternatives can be heard. What can be heard is a myopic, self-referential construction we call the Western system. The ability to contextualize the development of tools has powerful implications for understanding the limitations of these system tools (Jackson 2000). The second tenet, methodological pluralism, broadly refers to using different methodologies in combination with one another rather than strictly utilizing one approach or set of tools. Pluralism has several important benefits in critical systems thinking, one of the most relevant being that critique in recent decades has emerged about scientific disciplines and their notion for single, totalizing approaches that demand an “objective” perspective, in which there is one singular truth. These two principles together are to be used in service of some notion of the third, improvement or emancipation. That is, that any approach to knowledge creation and problem solving, will inevitably, inadvertently, reinforce the existing power structures/relationships (Jackson 2000; Midgley 2000). Complex and critical systems thinking provides a unique set of conceptual tools or heuristics for understanding the dynamics and tight interrelationships between social and ecological systems. And it has also led some scholars to a provocative set of questions about the nature and role of science and, more generally, knowledge in socio-ecological systems (Maturana and Varela 1980, 1987; Midgley 2000).

In approaching complex, social–ecological problems from a systems perspective, much of the literature points to the application of several key elements of a systems approach. However, as complex issues of cultural diversity, identity politics, and reconciliation rightly come to the fore, discussing perspective and purpose may be inadequate to the task of understanding the true complexity of systems in the context of these important issues. Knowledge does not, of course, emerge in a vacuum and, aside from physical constraints, there are social or institutional constraints on how knowledge emerges. The very “systems” in which systems thinking emerged as a discipline have placed constraints on the applicability of these tools in more complex contexts, such as situations in which multiple worldviews are interacting with one another. Resilience and transformation scholars are recognizing the importance of using other ways of knowing to tackle complex problems, through tools such as multiple evidence-based approaches and bridging knowledge systems (Tengö et al. 2017; Lam et al. 2020). Goodchild (2021, p. 79) in her work on Relational Systems Thinking highlights some key distinctions between different forms of knowledge creation, especially in systems-based approaches, and invites “a more relational disposition to collaborative knowledge creation and sharing”. We believe that the concept of trans-systemics should be added to this list of systems tools for truly addressing the complexity of systems where vastly different frames of reference need to be brought to the fore.

## Thinking about thinking in systems: critical legal theory, echoes in gender, and ethnicity

To understand the journey of trans-systemics (and more specifically Indigenous trans-systemics), we must make a number of stops analyzing our legal systems. The overriding theoretical basis for this expedition is Critical Legal Studies (CLS) which states that the law is necessarily intertwined with social issues. CLS believes that the foundations of law are created and maintained through biases which support the interests of those who create the law. In this way, law is a tool to favor those already advantaged and in power by maintaining the positioning of the underprivileged. CLS necessarily incorporates non-legal arenas, such as social theory, economics, philosophy, and politics:

Critical race theory not only dares to treat race as central to [] law and policy…. it dares to look beyond the popular belief that getting rid of racism means simply getting rid of ignorance, or encouraging everyone to “get along.” . . . .be sobered by the recognition that racism is part of the structure of legal institutions, but also to be invigorated by the creativity, power, wit, and humanity of the voices speaking about ways to change that structure. As race relations continue to shape our lives in the new century—setting the stage for new tragedies and new hopes—critical race theory has become an indispensable tool for making sense of it all (Angela Harris xix-xx, as cited in Delgado and Stefancic 2001).

CLS splintered into numerous subcategories. Feminist legal theory (FLT) examines gender and unequal representation within law (on the creation side). FLT further splinters as women of color articulate their lack of representation as FLT appears dominated by Caucasian female voices. People on the gender spectrum make similar declarations. Critical Race Theory, which examines the role of race in the law, has also fragmented as Black voices dominated the literature. Tribal Critical Race Theory addresses many of the same issues of subalternism, underrepresentation, and lack of voice.^3^

This continual splintering, fragmenting, and fracturing of the voices that challenge the system and its status quo create, what in resilience parlance we refer to as, poverty traps (Carpenter and Brock 2008), that allow the structures of the system to be maintained by the exact group these schools of thought are attempting to overthrow. By attempting to ensure that all voices are heard, these schools of thought become so specialized that they eventually have limited effect on the systems they are attempting to change. The voices for transformation and adjustment eventually become white noise and the system swings back to its original pool of actors. The agents within the system of law continue to hold control over the influencing of the system for their own benefit, creating laws that act on the Other.

## Trans-systemics

One group that is of particular interest to the authors and the subject of systems more generally is to be found at the McGill University’s Law School. McGill has created a methodology of reviewing and interpreting Canadian jurisprudence—trans-systemics—by looking at both ‘official’ languages and both ‘official’ methods of law (civil and common law). Situated in Montreal, Québec, which is a bilingual city and province with a bijural legal system, the local landscape offers practicality for teaching legal systems in a comparative way. The private law of Quebec, which is drawn from the civil law tradition, interacts with a system of public law that traces its origins to the English common law tradition. What makes this discourse of particular interest is that it is dominated by French Caucasian jurists and academics using the language of the Subaltern to articulate their dissatisfaction at ‘not being heard’ in the common law dominated legal system.

Trans-systemics is a term coined by McGill to address the idea that there is a bijural legal system in Canada based on both English and French legal traditions. In looking at the Canadian legal context, it is clear that the common law system (derived from our English foundations) largely diminishes the voice of Canada’s French roots and the Civil Code by the magnitude of its power and influence over law in Canada. However, these two systems exist simultaneously in Canadian law, and McGill seeks to teach both of these in a comparative setting. In 1998, the Faculty of Law undertook the effort to offer an integrated comparative 3-year curriculum, known as the McGill Program, that teaches even first-year introductory courses, such as Contracts and Torts, from a comparative perspective. The ultimate aspiration of this program, however, is to transcend the fixation on the study of law as the study of “legal systems”—hence the label “trans-systemic” legal education (Dedek and de Mestral 2009). Since then, the faculty has employed a Trans-systemic approach to the legal education of its students enabling them to graduate with both a civil and a common law degree by studying “the world’s great legal traditions in an integrated fashion.” This makes sense given the location of McGill.

McGill’s Faculty of Law has naturally been teaching law in a comparative, bijural way for the past 4 decades. In a world of borderless human interaction, however, a localized legal education is insufficient. McGill’s unique Trans-systemic model of legal education ensures that students graduate with a cosmopolitan understanding of the law, one that is not confined to specific jurisdictions, or even legal traditions (Paul-André Crépeau Centre for Private and Comparative Law n.d.). McGill is generally recognized as the genesis of the Trans-systemic. The program suggests that students graduate with a cosmopolitan understanding of the law, that is not bound to a specific jurisdiction, jurisdictions, or even legal traditions. Trans-systemics claims to recognize “legal pluralism as a pervasive phenomenon in the modern world” (Morissette 2002).

However, the structure that McGill is bounded by is notably an autopoietic, positivist legal system with very high resilience (Teubner 1993; Luhmann 1995). Inherent within positivism is the acknowledgement that there is one singular way to derive knowledge in the world and everything must fall within those definitions. Inherent within this ideology are concepts that we, as a society, so take for granted that we are blind to our prejudices. Using this control of ‘proper’ signifiers, such as the requirements for laws to be written to be considered legitimate, we discount aspects (or entire systems) that do not look ‘right’. This is seen in McGill Law School’s disregard for anything that does not look like a Eurocentric positivist legal structure. The definition of trans-systemics used by McGill demands the recognition of another system (Civil Code) while denying the existence of any other systems that are present in Canada.

Not only does McGill’s model deny the existence of other systems, but it ironically ignores the history of the City of Montreal. Montreal, Québec falls under the Montreal Treaty 1701 (aka Montreal Convention or The Great Peace of Montreal). The Montreal Treaty was a peace agreement with the Iroquois Confederacy (also known as the Five Nations Confederacy). In July 1700, delegates from four of the Iroquois nations (the Mohawk were absent) met with Governor Callière of Montréal to inaugurate peace talks with the French and their allies. Thirty nations sent a total of 1300 delegates to discuss over several weeks, during which gifts were exchanged and the accords were signed. To this day, over 300 years later, trans-systemics at McGill only recognizes two legal systems, and neglects to acknowledge the Indigenous systems which allowed for the creation of New France and Québec.

From an Indigenous Trans-systemic perspective, the issue is not that one of the two voices that make up Canada is not being heard, but rather that 30 of 31 jurisdictions are not being heard. The civil and common laws are both Eurocentric products that were imposed and deny Indigenous ways of knowing. McGill’s definition of trans-systemics enables us to consider multiple systems overlapping with one another, but it limits which systems are included in this consideration. Taking into consideration the limitations of systems thinking and critical systems when exploring contexts with multiple worldviews interacting with one another, the next section of this paper will introduce the concept of trans-systemics as a “tool” or solution for navigating these challenges.

### Indigenous trans-systemics

Building on the concept of legal trans-systemics from McGill and integrating insights from critical systems theory, our version of Indigenous trans-systemics poses new questions about the nature of systems, the resilience of conventional systems, developing knowledge about them, and structural barriers to “hearing” existing systems, such as Indigenous legal, linguistic, governance, and knowledge systems in the context of colonial systems. Much work has been done to discuss processes of decolonization, Indigenization and reconciliation, especially in recent years in countries such as South Africa, Canada, Australia, and New Zealand (for example, Kovach 2005; Smith 1999). All of this work points to the need to understand the profound impacts of colonization on Indigenous nations around the globe and rethink how, for instance, research is undertaken with Indigenous groups as well as making space for Indigenous ways of knowing in the academy, Indigenous legal systems, Indigenous languages, and Indigenous governance systems in the context of colonial systems.

The concept of Indigenous trans-systemics contributes to this growing body of literature and practice by integrating the Eurocentric, scientific concept of a “system” and ways of developing knowledge so that the ontological and epistemological assumptions that Eurocentric scientists and policymakers continually make are brought to the surface. This occurs by highlighting the autopoietic and bounded aspects of systems, and therefore requiring a critical approach to exploring knowledge creation around systems. Autopoietic notions of systems in combination with critical systems approaches help to explain why colonial, Eurocentric thinkers often cannot see Indigenous systems, as they are starting from a bounded, self-referential paradigm and therefore need to be more critical of their ontology and epistemology when attempting to meaningfully engage with Indigenous collaborators. In this way, Indigenous trans-systemics is a heuristic to aid in reflexivity and humility when attempting to make space for Indigenous ways of knowing and being.

If we return to the legal context and consider the inclusion of Indigenous laws in the Canadian legal context, the distinction between Aboriginal and Indigenous laws becomes important. Aboriginal laws (such as the anti-Potlatch laws) were typically created and imposed to obfuscate and eliminate the existing Indigenous property laws that were firmly ensconced in societies prior to European contact. These Aboriginal laws are actually Canadian laws applied to Indigenous people, communities, and nations. Indigenous law is the rules, protocols, procedures, limitations, and deterrence that grew from the territory to guide the original inhabitants. Due to centuries of colonization and assimilation, the volume on, or the resilience of, these Canadian legal systems is turned up so loud that it becomes almost imperceptible to hear the large number of Indigenous legal systems. Canada believes that Indigenous systems have been included by simply subsuming them into Canadian Eurocentric structures, when in fact the opposite is the case. In this context, not only is it important to be aware of the volume of the different systems at play, but it is also imperative to consider how we might turn down the volume of the loudest, most dominant and resilient systems to allow others to be heard.

### Moving trans-systemics beyond legal definitions

Although developed in a particular legal context, the principles behind the terminology may be used to expand the discourse and look at alternate systems within the Canadian context and how they interact with each other. Trans-systemics, in a more general sense, demands we look beyond the system in which Canada is comfortably inculcated. We must turn up the volume of the other understandings of systems. This includes how we do business, interact with our environment, and how we educate.

From an Indigenous perspective, trans-systemics also does not need to be considered strictly in the Canadian legal context. The Indigenous worldview suggests law is not a system in and of itself. Indigenous worldviews provide a more holistic, all-inclusive, meta-system perspective. Law is part of our storytelling and literatures, but is also part of economy, art, work, family, and politics. These systems cannot be bound within a perspective, field, or rationality. Within many Indigenous philosophies, plants, animals, minerals, and waters are animate, have a claim to inclusion in the system, and all decisions must take their needs into consideration. Further, personal worth is determined by the gifts and service you provide to others rather than what one has accumulated. These ideas probably sound foreign to many readers. This is because the Canadian system has denied these law perspectives within our legal system, in effect muffling Indigenous aspects of the discourse. Because these articulations do not fit into the highly resilient status quo they are silenced, minimized, or denied.

To understand this trans-systemics discourse, we can revisit the idea of volume levels of overlapping systems. If we imagine a car as a larger system, it is comprised of a number of smaller systems, including fuel, electrical, exhaust, steering, and the stereo. At some point, the car begins to require maintenance and repairs, but it is too costly to fix. The car starts making a noise while driving or sitting idle which is likely a symptom of a much larger problem, but since it is expensive, the easiest solution is to turn up the stereo to ignore the noise. One will go so far as to purchase a new stereo, buy louder speakers, install more powerful amps, anything to avoid what was actually wrong with the system. Trans-systemics is an attempt to help surface these deeply entrained, unconscious and problematic patterns of thought and behavior. The stereo system is so loud we can deny the larger problems in the engine system. But what is the first thing a mechanic does when you drive that car into a garage? They turn down the stereo system, so that they can hear the other systems and identify any problems. The stereo system is not necessary for the underlying system to run. Trans-systemics is the ability to turn down the stereo on the car to hear the other noises being made from the other systems at play.

### An Anishinabe trans-systemic view of social–ecological systems

To provide greater clarity regarding Indigenous systems, consider the Anishinabe view of systems. More than just including everything within the system as part of the system, Indigenous trans-systemics does not recognize the role of the passive observer. Rather, the individual attempts to find their place within the system. There is no role for an “objective observer” of a social–ecological system. Within the Anishinabe system, the individual is responsible to the system and for the system. Acknowledging one’s place within the system, one is obliged to every other member of the system. If you can see the system, the system is affecting you and you are a part of that system. As you begin to describe the system, it is essential to include your influence or the effect the system is having on you. It is inherently relational (Goodchild 2021). The Eurocentric discussion around systems never describes the role that we as observers have on the system. It is a false assumption made in the conventional systems thinking that our perception of the system does not have any influence over the system. The notion of objectivity and neutral observation does not fit within the Anishinabe understanding of systems.

In the book The Gift, Mauss (1966) looks at a number of Indigenous systems and finds these ‘archaic’ societies all have an understanding of individual as associated or attached to the system to which they have become a part. Your presence within the system is seen as a gift, as are the relationships that tie you to the other members of the system. Stories are created and shared to better understand the relationships in which the individuals are engaging. Gifts would be exchanged to show the reverence and respect each party has for the other parties and the relationship itself. Among the Anishinabe, this understanding of the relationships and the gifting is continuously re-establishing the agreements as part of the system itself was the standard practice. The Anishinabe offer the gift of invitation into their community. On choosing to accept this offer (the initiation into the community), one accepts the responsibilities that go with it. Being recognized by the Indigenous system establishes a relationship that obligates the individual to the community, the collective individuals within the community, and to the gift itself. This idea is referred to as total prestation by Mauss. These obligations take the form of reciprocal expectations on every individual within the community. The acceptance of the gift creates a communitarianism relationship. This understanding of the relationship of the individual is always as a part of the Anishinabe system. The Eurocentric idea that to be rational, one must be apart from the system is foreign to Anishinabe understandings of what the system is itself.

## Conclusion

Systems are loud. Many of us may not even be aware of the noise, since we have never known how to control the volume. This paper is intended to explore opportunities to turn down the volume of how we understand systems and offer new perspectives on what it means to define and examine a system and a system’s resilience to change.

When exploring systems, the first step is to consider perspective, components, boundaries, and structure, as well as resilience of a system of interest. These “building blocks” form the basis of our understanding of the structure and dynamics of systems that we move within. As our world becomes increasingly interconnected and complex, we must also start considering perspective and purpose with a critical lens. We need to consider who is describing the system, through what perspective, and what implications that might have on the overall description of the system. This is part of a power dynamics where the describer, the described, and the vantage point of the description, all point to a validation of ‘the system’ that may not only be to the detriment of the Indigenous systems being described, depicted, and defined as inferior, archaic, or extinct but also to the systems that allow this narrow view to limit the potentials within themselves. Using a critical systems approach is often done so in the context of emancipation or improvement, which requires acknowledging that there are some injustices or issues in the way the system is currently being understood or described.

A logical extension of critical systems thinking is the notion of a Trans-systemic approach to describing and intervening in systems. This approach requires us to consider that there are multiple overlapping systems in place simultaneously. With origins in legal academics, McGill Law School has formalized the idea that multiple systems can be in place simultaneously and can co-exist when properly acknowledged. The shortcomings of McGill’s definition are that it focuses on legal systems, and incorrectly assumes a bijural system in Canada. The reality of the Canadian landscape is that there are dozens to hundreds of legal systems that are not being considered due to hundreds of years of colonization and oppression. The reader may be asking themselves, is the inclusion of a completely different way of viewing systems provided by Indigenous trans-systemics for the benefit of Eurocentric systems that need to hear the possibilities available in Indigenous systems, or to assist those Indigenous systems in being heard, considered, and included to sustain these systems for Indigenous generations to come? The answer is yes. All benefit from inclusion and expansion of the systems at play. All agree that to hold our structures in an unchanging cycle invites system death.

We would like to offer the following three key insights from trans-systemics:

1. Trans-systemics is a call to humility in social–ecological systems thinking. It suggests that we “turn down the volume” of the system we recognize and offer an opportunity for others to be heard. Trans-systemics is an invitation for researchers and practitioners to engage in the difficult inner work of building the capacity to sit in complex contexts and not be overwhelmed (Machado de Oliveira 2021) and to engage in a form of self-psychoanalysis—acknowledging our different and deeply entrained worldviews and become more relational in our thinking about knowledge creation and sharing (Goodchild 2021). Trans-systemics requests we move beyond reflexivity and acknowledge that we unconsciously apply a way of thinking about systems that is the result of the structure of our brains, the history of our psyches, and the long-standing institutions that reinforce and reward those patterns of our thinking.
2. We all suffer from bounded rationality and are limited by our conceptual, theoretical, and philosophical lenses. Indigenous trans-systemics offers a new way of engaging and understanding systems that denies the purported autopoiesis of any social system, instead recognizing and searching out the system’s interdependence.
3. A Trans-systemic approach is an invitation to reflect on one’s assumptions, biases and priveleges, honesty regarding one’s limitations (both perceived and unperceived) and accepting the resultant humility when one is confronted with work across very different perspectives (new truths). Rather than attempting to fit Indigenization or Reconciliation into our current inadequate mindset, trans-systemics processing requires deep, systemic change which will transform our conceptions of systems. Audre Lorde wrote, “…the master’s tools will never dismantle the master’s house. They may allow us temporarily to beat him at his own game, but they will never enable us to bring about genuine change. And this fact is only threatening to those…. who still define the master’s house as their only source of support” (1983, p. 112). This change will be scary as it is how we derive our worth, our income, our very meaning. Institutions currently attempting to reconcile these processes must alter the presumptions, processes, accommodations, and objectives inherent in an unconscious understanding of social–ecological systems. It refuses to accept symptomatic or tokenistic fixes, instead demanding new, foundational solutions.

In most contexts, there tends to be a single, highly resilient system that dominates the discourse, often from the Eurocentric worldview. Trans-systemics asks us to turn down the volume on the systems we assume to be universal to allow other systems to be heard. In doing so, we move a step closer toward acknowledging that there are multiple systems interacting with one another simultaneously, and that there is more than one way of looking at a particular system or issue. Trans-systemics offers new opportunities for tackling issues related to complexity, reconciliation, and the simple recognition that the loudest systems are not necessarily the best or the most equitable. Our discussion on Indigenous trans-systemics offers a new way of engaging and understanding systems in the hopes that the field of systems thinking can become more diverse and open to other ways of knowing.

Using Indigenous understandings of systems as expressed in the Anishinabe story of Nahnabush, we can see that the role of the observer is to dive into the system. There can be limited understanding on the periphery of systems. We cannot assume a role of rational, emotionless witness. Witnessing is not passive. It is active and the system cannot help but be affected by the presence of a new attractant within the waters. According to Indigenous storying, one has to be part of the system, changed, engulfed, encompassed, and transformed by it, to have the right to speak to it. Any observation of another’s system is folly. The act of observation alters the observer and the system. It is not the same as before contact. This perspective, found in an Anishinabe story that is thousands of years old, speaks to a very different perspective than the one we teach in the academy. One final takeaway from this Indigenous story as Indigenous systems theory can be seen in being of Nahnabush as Trickster and the Tricksters role with the story. Very often, Trickster enters the system, is transformed, and transforms, and leaves. The story does not end. Just as the story does not end for the lack of Trickster’s presence, systems are always in place, functioning, long before ‘outsiders’ came to ‘observe’ them. And they continue well after everyone has finished looking and left the territory. Indigenous Trickster stories can teach us a new way of looking at systems and theories of systems. We just need to listen.

## Data Availability

No data availability statement for this article.

## Footnotes

1 The standard indoctrination that occurs in Canada’s education system and the volume it maintains is fodder for another paper looking Indigenous trans-systemics with Canada’s education systems. There is no room in this paper to move into a proper articulation of the issues within that system. We welcome these additions to the narrative.

2 T. Brodhead (personal communication, May, 2019) at a gathering of Social Innovators.

3 According to Bryan Brayboy TribalCrit theory is based on a number of tenets “1) Colonization is endemic to society; 2) U.S. policies toward Indigenous peoples are rooted in imperialism, White supremacy, and a desire for material gain; 3) Indigenous peoples occupy a liminal space that accounts for both the political and racialized natures of our identities; 4)Indigenous peoples have a desire to obtain and forge tribal sovereignty… and self-identification; 5) The concepts of culture, knowledge, and power take on new meaning when examined through an Indigenous lens; 6) Governmental policies and educational policies toward Indigenous peoples are intimately linked around the problematic goal of assimilation; 7) Tribal philosophies, beliefs, customs, traditions, and visions for the future are central to understanding the lived realities of Indigenous peoples, but they also illustrate the differences and adaptability among individuals and groups; 8) Stories are not separate from theory; they make up theory and are, therefore, real and legitimate sources of data and ways of being; and 9) Theory and practice are connected in deep and explicit ways such that scholars must work towards social change” (Brayboy 2005, pp. 429–430). See also Davidson et al. (2018), Mackey (2015), Paris (2012), and San Pedro (2015).
